# New synchronization method for *Plasmodium falciparum*

**DOI:** 10.1186/1475-2875-9-170

**Published:** 2010-06-17

**Authors:** Lisa C Ranford-Cartwright, Abhinav Sinha, Georgina S Humphreys, Jonathan M Mwangi

**Affiliations:** 1 Division of Infection and Immunity, Faculty of Biomedical and Life Sciences, University of Glasgow, Glasgow Biomedical Research Centre, 120 University Place, Glasgow G12 8TA, Scotland

## Abstract

**Background:**

*Plasmodium falciparum *is usually asynchronous during *in vitro *culture. Although various synchronization methods are available, they are not able to narrow the range of ages of parasites. A newly developed method is described that allows synchronization of parasites to produce cultures with an age range as low as 30 minutes.

**Methods:**

Trophozoites and schizonts are enriched using Plasmion. The enriched late stage parasites are immobilized as a monolayer onto plastic Petri dishes using concanavalin A. Uninfected erythrocytes are placed onto the monolayer for a limited time period, during which time schizonts on the monolayer rupture and the released merozoites invade the fresh erythrocytes. The overlay is then taken off into a culture flask, resulting in a highly synchronized population of parasites.

**Results:**

Plasmion treatment results in a 10- to 13-fold enrichment of late stage parasites. The monolayer method results in highly synchronized cultures of parasites where invasion has occurred within a very limited time window, which can be as low as 30 minutes. The method is simple, requiring no specialized equipment and relatively cheap reagents.

**Conclusions:**

The new method for parasite synchronization results in highly synchronized populations of parasites, which will be useful for studies of the parasite asexual cell cycle.

## Background

The human malaria parasite *Plasmodium falciparum *is usually asynchronous during *in vitro *growth [1,2], with all asexual stages of the parasite present. The generation of cultures containing highly synchronized parasites is necessary for studies of the cell cycle, for example, allowing accurate measurement of the lengths of different phases of the parasite life cycle.

Various synchronization methods have been published, which rely on removal of different parasite stages by differential osmotic lysis [3], physical separation relying on differential density [4-6] or by magnetic separation [7], temperature cycling [8], or cell cycle inhibitors [9]. Reviews of the available synchronization methods and their advantages and disadvantages have been published previously [9,10]. However all of these methods produce a population of parasites with a relatively wide age range - the lowest reported is in the range of 3-5 hours [6,10]. An acknowledged problem is that narrowing of the range of age results in a reduction of parasitaemia.

A new method of synchronization has been developed by combining a recently published method to enrich cultures for later stage parasites using Plasmion (Laboratoire Fresenius Kabi, France) [11], with an inverted version of the "plaque assay" of J. Williams [12]. The enrichment method [11] is based on the slower sedimentation rate of late trophozoites and schizonts from K+ (knob-expressing) strains [13] though a gelatin solution (Plasmagel), thus allowing their separation from earlier parasite stages and from uninfected erythrocytes [5]. Plasmion, a plasma substitute used in hospitals for hypovolaemia, is used in place of Plasmagel, which is no longer widely available [11]. This method allows the collection of merozoites within a user-specified window that can be as little as 30 minutes, or even less. The resultant culture contains infected erythrocytes with a very narrow age range, making this method very suitable for studies on cell cycle.

## Methods

### Enrichment of late trophozoites and schizonts from *in vitro *cultures using Plasmion (Figure 1)

**Figure 1:**
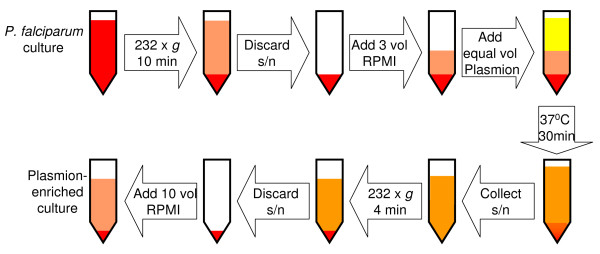
**Schematic of Plasmion enrichment**.

Asexual cultures of *P. falciparum*, grown according to standard protocols, were subjected to Plasmion treatment as previously described [11]. Typically, a culture was chosen with a relatively high proportion of later stage parasites with a parasitaemia of between 3 and 10%. The culture material was centrifuged to pellet the red blood cells (232 × *g*, 10 minutes), the supernatant was removed, and the pellet was resuspended in fresh culture medium at a ratio of 3:1 medium: pellet. An equal volume of Plasmion (Laboratoire Fresenius Kabi, France) was added and the solution mixed and incubated at 37°C for 30 minutes. After incubation, the supernatant containing the older stages of the parasite (schizonts and trophozoites) was removed to a fresh tube, and the pellet containing the uninfected red blood cells and ring stage parasites was discarded. The collected supernatant was then centrifuged (232 × *g*, 4 minutes) to pellet the parasites, and the supernatant was discarded. A thin blood smear was made from the resultant pellet to measure the parasitaemia and to identify the parasite stages present. The cellular pellet was then resuspended in 1:10 pellet: incomplete medium (RPMI without serum), to give approximately 10% packed cell volume (PCV) for use on the monolayers.

### Formation of monolayers of parasitized erythrocytes using concanavalin A (Figure 2)

**Figure 2:**
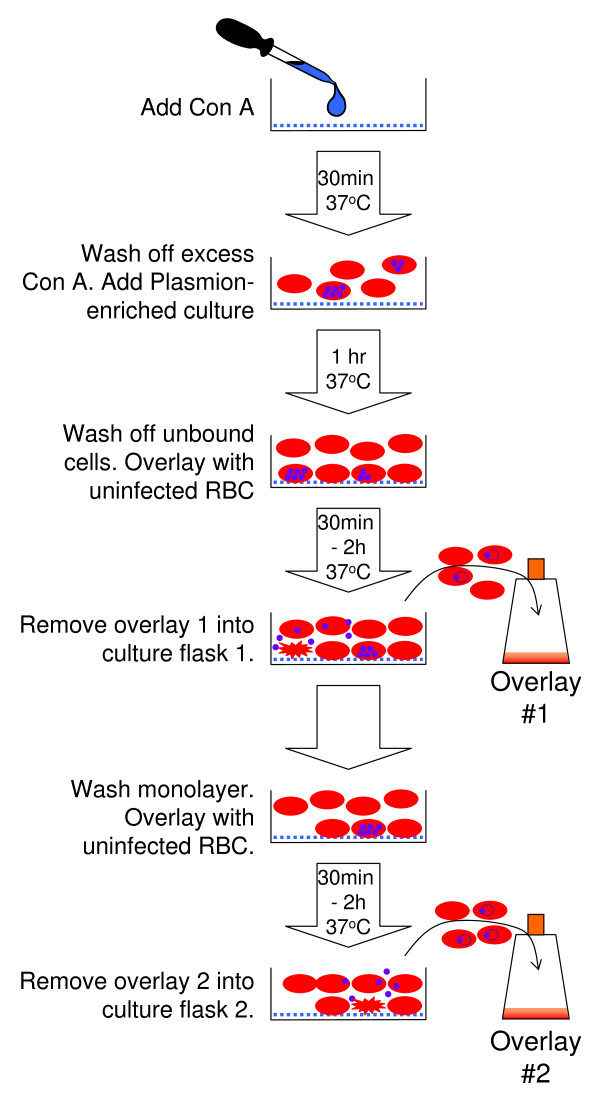
**Schematic of collection of synchronized ring stages from parasitized erythrocyte monolayers**.

1.5 ml of 10 μg/ml solution of concanavalin A (Sigma) was added to a sterile polystyrene tissue culture dish (diameter 35 mm) and incubated for 30 minutes at 37°C [12]. Excess concanavalin A was removed and the dish washed with incomplete medium (RPMI without serum). 1.5 ml of the Plasmion-enriched *P. falciparum *culture was added to the dish, placed into a modular incubator (Billups-Rothenberg, Inc), gassed (96% N_2_: 3% CO_2_: 1% O_2_) and incubated for 1 hour at 37°C to allow the erythrocytes to adhere to the concanavalin A.

Unbound erythrocytes were removed by aspiration and the monolayers were washed 3-5 times with incomplete RPMI medium to remove any unbound cells. The resultant monolayer contained late trophozoite- and schizont-infected erythrocytes immobilized onto concanavalin A.

### Collection of synchronized ring stages from parasitized erythrocyte monolayers (Figure 2)

1.5 ml of uninfected erythrocytes (10% PCV in complete RPMI) was laid over the monolayer, and the dish was then returned to the modular incubator, which was gassed and incubated at 37°C for the required time period (e.g. 30 minutes - 2 hours), depending on the range of ages required in the resultant culture. After this time the overlay cells were resuspended by gentle swirling, and collected and placed into a 25 cm^2 ^tissue culture flask. Smears were taken from this flask immediately ("Time 1 hour" smear), and stained with Giemsa. 1 ml of complete medium was added and the culture gassed as before, and incubated at 37°C.

The monolayer was then washed 3-5 times with incomplete medium to remove any unbound cells, and was incubated with a further 1.5 ml of uninfected erythrocytes at 10% PCV for the next hour. "Overlay 2" cells were collected after this period as before.

Repeated overlays could be applied to the same monolayer over the next 12 hours, and ring stage parasites collected over varying time periods to establish cultures of different ages and parasitaemia.

The "Time 1 hour" bloodsmear from each of the overlays was examined as soon as possible. Where there had been no or limited release of merozoites from mature schizonts in the incubation period, the resultant overlay culture had no or low ring stages present, and was discarded. The "Time 1 hour" smear was also discarded if there were parasites of any stage except for young ring stages. Such parasites probably indicate incomplete removal of unbound cells by the washing steps before incubation with uninfected erythrocytes.

## Results

### Enrichment of late trophozoites and schizonts from *in vitro *cultures using Plasmion

Plasmion treatment of asexual cultures produced an overall increase in parasitaemia of 4.2- to 7.7-fold (mean 5.8-fold) (Table 1). Both trophozoites and schizont stages were significantly enriched following Plasmion treatment, with a 6.0- to 14.3-fold increase in schizonts (mean 9.9-fold), and a 9.7- to 22-fold increase in trophozoites (mean 13.4-fold enrichment). A similar enrichment was seen for cultures of four different *P. falciparum *cloned lines (3D7, HB3, X12 and XP5).

**Table 1:** Plasmion enrichment of trophozoites and schizonts in *P. falciparum *cultures

Parasite	Parasitaemia before Plasmion (%)				Parasitaemia after Plasmion (%)				Enrichment factor			
	
	Total	R	T	S	Total	R	T	S	Total	R	T	S
3D7	**11.3**	6.1	2.4	2.8	**47.2**	2.8	26.1	18.3	**4.2**	0.5	11.1	6.5
3D7	**12.1**	6.9	3.1	2.1	**51.2**	2.5	35.9	12.8	**4.2**	0.4	11.6	6.0
HB3	**9.8**	4.8	2.6	2.4	**62.6**	2.1	26.3	34.2	**6.4**	0.4	10.1	14.3
HB3	**10.0**	5.0	1.0	4.0	**70.0**	1.4	16.0	52.6	**7.0**	0.3	16.0	13.1
XP5	**13.0**	6.0	3.0	4.0	**66.8**	2.8	29.0	35.0	**5.1**	0.5	9.7	8.8
X12	**10.2**	4.0	1.1	5.1	**78.1**	2.1	22.0	53.9	**7.7**	0.5	22.0	10.8
Mean enrichment									**5.78**	**0.4**	**13.4**	**9.9**

Asexual culture parasitaemia was estimated by examination of 2,000 erythrocytes for each culture, either immediately before or following enrichment with Plasmion. The numbers of ring (R), trophozoite (T) and schizont (S) stages present in each smear were also recorded. Trophozoites were distinguished from ring stage parasites by the appearance of pigment. Schizonts were distinguished from trophozoites by the presence of two or more nuclei. The enrichment factor was calculated by dividing the post-Plasmion treatment parasitaemia by that at pre-treatment.

### Collection of synchronized ring stages from parasitized erythrocyte monolayers

**Figure 3:**
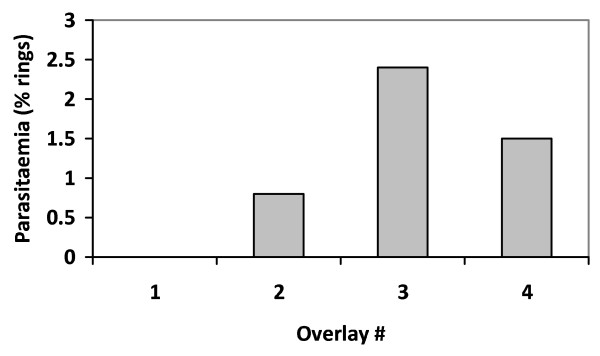
**Example of overlay culture from one monolayer**. A monolayer made from a Plasmion-enriched culture (XP5) at 66.8% parasitaemia was overlaid with uninfected cells forming overlay#1 for 1 hour. This was removed and replaced with fresh uninfected red cells for a further hour (overlay#2), and then with a third replacement (overlay#3) and finally a fourth (overlay #4) for a final hour. There was no detectable parasitaemia in overlay #1. Peak parasitaemia was observed in overlay #3. Only ring stage parasites were seen in overlay #2 to #4.

Overlay cultures containing ring stage parasites only were obtained from the majority of monolayers, indicating successful synchronization. An example of a single monolayer and four overlay cultures is shown for parasite clone XP5 in Figure 3. The overall starting parasitaemia of the culture used to generate the monolayer, following Plasmion enrichment, was 66.8% (35% schizonts: 29% trophozoites: line 5 in Table 1). This monolayer yielded a ring stage parasitaemia of 0.0%, 0.8%, 2.4% and 1.5% in successive one hour overlays beginning immediately after the establishment of the monolayer, indicating that there was no lysis of mature schizonts for first hour after the monolayer establishment, followed by a peak of schizont lysis 3-4 hours post monolayer establishment. The rings present in these one-hour cultures represent merozoites emerging from schizonts and reinvading fresh red cells within a one hour window, and are thus highly synchronized.

## Discussion

A simple new method is described to obtain highly synchronized asexual cultures of *P. falciparum *suitable for studies of the asexual cell cycle. Plasmion treatment [11] of the asynchronous culture increases the overall parasitaemia approximately six-fold, by the removal of uninfected and ring-stage infected red blood cells, and substantially enriches the culture for schizonts (approximately ten-fold) and trophozoites (approximately 13-fold). An overall decrease of ring stage parasites was also observed (Table 1). The efficiency of the method does not require starting cultures with high numbers of more mature parasites.

The enriched culture is used to create a monolayer of infected cells bound to a plastic Petri dish using the lectin concanavalin A. In the original description of the "Plaque assay" [12], uninfected erythrocytes are used to form the monolayer, and the overlay consists of parasitized red cells. Merozoites released from the overlay invaded the monolayer. Here the method has been inverted, forming the monolayer from parasitized erythrocytes, and the overlay of uninfected red cells. Mature schizonts in the monolayer release their merozoites which invade uninfected red cells in the overlay. The overlay is then removed to establish new asexual cultures. By limiting the time of contact between the monolayer and overlay, merozoites invade within a narrow time-window, which can be as short as 30 minutes, and could theoretically be shorter than this. The overlay culture thus represents a highly synchronized population of parasites.

The degree of synchronicity of the overlay culture can be changed by varying the length of time the overlay and monolayer remain in contact. Longer periods of time generally result in a higher parasitaemia, but with a less tightly synchronized parasite population. The initial Plasmion treatment increases the monolayer parasitaemia to greatly in excess of that normally possible *in vitro*, producing a higher parasitaemia in the overlay culture than would be possible without the use of enrichment. Thus it is possible to narrow the age range while maintaining a useful parasitaemia.

The chief difficulty with the method is to judge when the overlay should be placed onto the monolayer, at the period of maximum schizont rupture. The appropriate time can be estimated from the number and size of schizonts in the smear taken of the Plasmion-treated material. A single monolayer can be used to collect multiple overlay cultures (the maximum prepared is five one-hour overlays, but theoretically many more could be taken). In addition, in this paper a 2.6 cm diameter dish was used, but larger dishes could be substituted if larger volumes of synchronized culture were required.

In comparison to other available methods of synchronization, the new method is simple and cheap, requiring no specialized equipment such as magnetic columns [7] or complicated density gradients [14].

## Conclusions

By combining two existing techniques, a new method is presented that allows very tight synchronization of cultures compared to other published methods, down to a time window of 30 minutes (or less) if required.

## Competing interests

The authors declare that they have no competing interests.

## Authors' contributions

LCRC conceived the technique, designed the study, participated in the data analysis and drafted the paper. GSH optimized the Plasmion conditions and reviewed the manuscript critically. JMM and AS optimized and carried out the monolayer-based synchronizations, participated in the analysis of the results and reviewed the manuscript critically. JMM helped to draft the final manuscript. All authors read and approved the final manuscript.
